# A Method for Image Anomaly Detection Based on Distillation and Reconstruction

**DOI:** 10.3390/s23229281

**Published:** 2023-11-20

**Authors:** Jiaxiang Luo, Jianzhao Zhang

**Affiliations:** School of Automation Science and Engineering, South China University of Technology, Guangzhou 510641, China; 202121018689@mail.scut.edu.cn

**Keywords:** image anomaly detection, knowledge distillation, autoencoder

## Abstract

Image anomaly detection is a trending research topic in computer vision. The objective is to build models using available normal samples to detect various abnormal images without depending on real abnormal samples. It has high research significance and value for applications in the detection of defects in product appearance, medical image analysis, hyperspectral image processing, and other fields. This paper proposes an image anomaly detection algorithm based on feature distillation and an autoencoder structure, which uses the feature distillation structure of a dual-teacher network to train the encoder, thus suppressing the reconstruction of abnormal regions. This system also introduces an attention mechanism to highlight the detection objects, achieving effective detection of different defects in product appearance. In addition, this paper proposes a method for anomaly evaluation based on patch similarity that calculates the difference between the reconstructed image and the input image according to different regions of the image, thus improving the sensitivity and accuracy of the anomaly score. This paper conducts experiments on several datasets, and the results show that the proposed algorithm has superior performance in image anomaly detection. It achieves 98.8% average AUC on the SMDC-DET dataset and 98.9% average AUC on the MVTec-AD dataset.

## 1. Introduction

This problem can theoretically be reduced to image anomaly detection; that is, learning, establishing a pattern of normal image samples, and judging anomalies based on the difference between the pattern of the test sample and the pattern of the normal image. Deep learning can learn and represent complex abnormal features from a large amount of data and is currently the mainstream method used for image anomaly detection [1]. Following the development of convolutional neural networks, a rich variety of models and methods have emerged for different application needs, such as ResNet [2], Generative Adversarial Networks (GAN) [3], Normalized Flow models [4], Knowledge Distillation methods [5], etc. These methods provide many novel approaches to the problem of image anomaly detection, among which data reconstruction and distillation learning have received the most attention.

Data reconstruction methods usually use autoencoders (AE) as the basic structure. These structures project the original data to the low-dimensional feature space, reconstruct the data, and evaluate the anomaly based on the difference between the reconstructed data and the original data [6,7]. These methods aim to compress the information by using a bottleneck design, assuming that abnormal samples have worse reconstruction quality than normal samples. However, this assumption is not consistently upheld, as abnormal samples can also be reconstructed in some cases, making it difficult to distinguish them from normal samples. The main problems are that (1) the reconstruction target is dimension reduction rather than anomaly detection, and the feature representation is not optimized for anomaly detection [8]; (2) weak anomalies are similar to the background in appearance and difficult to detect based on the image difference. To solve these problems, this paper introduces the use of feature distillation to train the encoder, giving the latent variable space encoding significant discriminability in the feature space and obtaining the ability to detect anomalies at the feature level after distillation learning. By comparing the feature differences between the teacher network and the student network, weak anomalies that are difficult to find at the image level can be found at the feature level.

Methods based on knowledge distillation have also achieved good results in the field of anomaly detection [9,10,11]. These methods take the pre-trained convolutional network as the teacher network and then take a new convolutional network as the student network. The student network learns the representation of the teacher network using normal data. For abnormal data, the output of the two networks may produce a large difference, thereby enabling detection of abnormalities by measuring the information difference between the student network and the teacher network. However, the knowledge-distillation framework assumes that the student network will find it more difficult to reconstruct the features of anomalies. This framework thus has problems in common with autoencoder reconstruction: (1) this assumption is not satisfied for all types of anomalies, and the differences in feature expression for anomalies between the teacher network and student network might be minimal; (2) the distillation method cannot detect differences between the object to be detected and background noise and often generates abnormal responses to background noise. To address these issues, the authors of this paper first designed a dual-teacher network distillation strategy to reinforce the assumptions of the distillation method. This strategy makes it more difficult for the student network reconstruct the feature expression of anomalies. Secondly, the authors designed an attention mechanism that can extract the foreground mask of the object to be detected to filter out noise from the background. This approach can prevent performance degradation caused by background noise.

In order to improve the accuracy of image anomaly detection, the authors construct an AE algorithm for image anomaly detection based on distillation learning and introduce an attention mechanism to focus on the area of the detected object. This approach allows the rapid detection of various product appearance defect anomalies. The main contributions are as follows:

1. A distillation-based autoencoder structure

This structure uses feature distillation to train the encoder, thereby suppressing the reconstruction of the abnormal area. The autoencoder structure under feature distillation fully combines the semantic information at the feature level and the structural information at the image level, thereby improving the performance of the autoencoder in anomaly detection.

2. A feature distillation learning method under the dual-teacher network

This method forces the student network to learn from both teacher networks at the same time. The expression of abnormal information that has not been trained is more easily distinguished after learning from both teacher networks. This approach thus helps to improve anomaly detection by the feature distillation model, and weak anomalies can be more easily recognized.

3. Attention mechanism based on target foreground extraction

In order to allow the anomaly detection model to focus on the product to be detected, this paper proposes an attention mechanism based on target foreground extraction. This mechanism, when used for object class anomaly detection, can focus the model’s attention on the object, thereby filtering out irrelevant background interference.

4. Method for anomaly score calculation based on patches

This method compares the input image to the reconstructed image from the structure using different areas of the image, suppresses abnormal misjudgment arising from the edge response, and has high sensitivity to weak defects in the SMD capacitor.

This paper verifies the proposed image anomaly detection algorithm using the MVTec-AD [12] and SMDC-DET [13] datasets and obtains an average AUC score of 98.9% with the MVTec-AD dataset and an AUC score of 98.8% with the SMDC-DET dataset. The experimental results show that the proposed method has excellent results with both datasets, demonstrating the effectiveness and robustness of the algorithm.

## 2. Related Work

This section mainly focuses on the current status of research on image anomaly detection. Mainstream image anomaly detection methods are mainly dedicated to solving the difficult problem of defect detection in products in industrial production.

1. Reconstruction based on AE

Autoencoders are common unsupervised learning tools that extract useful features by learning to reconstruct input data. This method is widely used in anomaly detection because abnormal samples usually produce larger errors in the reconstruction process.

Work on this type of method is usually focused on the design of the encoder-decoder model and the definition of the algorithm for reconstruction quality anomaly scoring to avoid the above situation. In of the design of encoding decoding models, ongoing work mainly focuses on in-depth research on reconstruction strategies and latent variable-space constraints. One published approach [14] integrates three different types of autoencoders and fuses their features to detect anomalies. Other approaches [15,16] assign different weights to components of different frequencies to improve the differentiation of positive and negative samples by the autoencoder. Another investigator [17] proposes the introduction of adversarial autoencoders (AAE) [18] to constrain the distribution of latent variables, making it to map abnormal samples to low-likelihood areas. The probability of abnormal reconstruction under the integrated model is lower. Another author [19] analyzes data from the low-dimensional vector space and uses clustering methods to classify the latent coding of the Deep Autoencoder (DAE) [20], thereby avoiding the problem of abnormal reconstruction quality. Still other studies [21,22] use memory mechanisms to suppress the expression of anomalies in reconstruction. MemAE [21] introduces a memory bank to store the latent variables of normal samples. During inference, the related memory in the bank is used for reconstruction to avoid reconstruction of the abnormal area. However, this method also affects the reconstruction of normal samples. There are studies [23,24] that propose a self-supervised strategy of image masking to destroy normal samples and force the autoencoder to repair the image, thereby allowing the autoencoder to gain the ability to recognize anomalies. The core purpose of these methods is to strengthen the assumption of AE: the reconstruction quality of abnormal samples is worse than that of normal samples. Unfortunately, such differences in reconstruction quality are not always apparent. This paper proposes an autoencoder structure based on feature distillation, which can comprehensively analyze possible abnormal areas in the input samples in the feature space and image space. The abnormal response information from the two is complementary. Even if the autoencoder reconstructs the abnormal area, the method proposed in this paper can still effectively detect the abnormal value.

To define the reconstruction quality anomaly score algorithm, methods based on reconstruction also focus on detecting differences between the input image and the reconstructed image. Many methods based on reconstruction generation [14,21,23] use the mean squared error (MSE) to measure the difference between images. However, the implicit assumption of MSE is that pixels are independent of each other, an assumption that ignores the dependence information between pixels and often cannot accurately distinguish between normal and abnormal image areas.

In order to resolve the performance constraints that arise from using MSE, AE-SSIM [7] introduces SSIM [25] to calculate the difference between images. SSIM measures the difference between images from three aspects: structure, brightness, and contrast. RIAD [23] extends GMSD [26] to propose a multi-scale variant method, MSGMS. GMSD measures the difference from the perspective of gradient similarity. Although MSGMS responds sensitively to slight anomalous it is also very sensitive to the edges of the image and is prone to misjudgment. In response to this problem, this paper proposes a method for anomaly score calculation based on patches to avoid the impact of edges.

2. Knowledge Distillation

Knowledge distillation is a model-compression technique that improves the performance of small models by having a student model mimic the behavior of a teacher model. In anomaly detection, knowledge distillation can be used to extract the teacher model’s understanding of normal samples and pass this knowledge to the student model.

Anomaly detection methods based on knowledge distillation [9,11,27,28] have shown excellent performance. For example, US [27] unifies the output scale between the teacher network and the student network through a decoder. MKD [9] constructs a small student network to learn the feature expression of the teacher network and introduces a multi-scale method to evaluate anomalies. STPM [28] uses a multi-scale feature pyramid strategy to enable the student model to obtain rich mixed knowledge and provides high-precision capabilities for anomaly localization. RD [11] combines the structural characteristics of the autoencoder and uses the bottleneck of the autoencoder to propose a reverse-distillation paradigm to improve anomaly recognition by the knowledge distillation model.

The optimization methods used by the methods based on knowledge distillation and the methods based on reconstruction have a commonality: it is necessary to increase the difference between the anomaly and the normal class at the feature/image level while maintaining consistency between the normal classes at the feature/image level. In response to this commonality, this paper combines the image reconstruction of the autoencoder and the feature expression learning of knowledge distillation and jointly optimizes from the feature and image levels to jointly evaluate the anomaly. In order to improve the sensitivity of the distillation model to anomalies, this paper proposes a distillation structure under the dual-teacher network. It will be more difficult for a student network to simultaneously reproduce the abnormal feature representations of two teacher networks.

3. Attention Mechanism in Anomaly Detection

For object class anomaly detection, the anomaly detection method based on knowledge distillation tends to confuse background noise with anomalies in the object itself. The interference noise that appears in the background will affect the detection performance of the model, thus requiring the introduction of an attention mechanism. The attention mechanism can help the model focus on the key parts of the input data, thereby improving performance in anomaly detection.

Most of the attention mechanisms proposed in the literature [29,30,31] utilize supervised tasks, but anomaly detection often emphasizes unsupervised tasks and needs to combine the characteristics of the model to allow the attention module to learn useful feature information. The attention mechanism in many anomaly detection methods is deeply bound to the proposed model [32,33,34,35]. CAVGA [32] generates attention maps through Grad-CAM [36], and the attention maps need to cover the entire normal image using normal data, expecting the model’s attention to focus on all normal areas of the image. When testing, if the attention value is low, it is considered to be abnormal. MAMA Net [33] proposes a multi-scale global spatial attention block (MGSA), which can overcome the problem of the limited receptive field of the convolution operator. RSTPM’s [34] attention module is obtained from the feature aggregation channel of the teacher network, thereby emphasizing or suppressing pixels. SAGAN [33] introduces the CBAM [29] module into each layer of the generator’s encoder to obtain the attention information from the corresponding layer. The purpose of the attention mechanism in this paper is to generate a mask that can highlight areas where anomalies need to be detected in the image, thereby removing irrelevant background interference and improving anomaly detection by the model.

## 3. Materials and Methods

**Figure 1:**
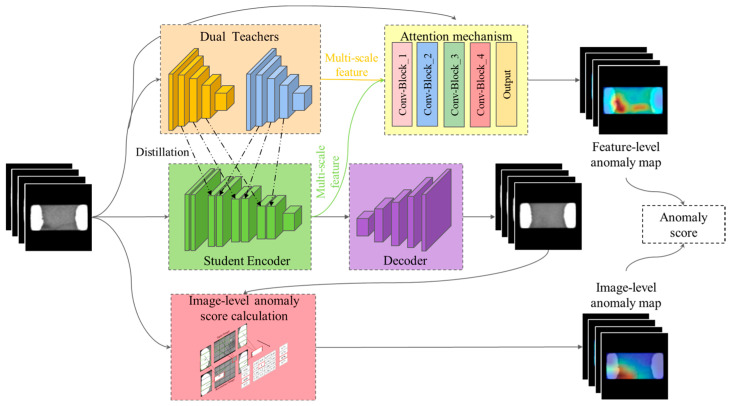
Flowchart of proposed method based on feature distillation and autoencoder structure.

The problem of image anomaly detection can be described as follows: suppose $I^{N}$ = {$I_{1}^{N},I_{2}^{N},\ldots ,I_{n}^{N}$} is a dataset containing normal images and $I^{T}$ = {$I_{1}^{T},I_{2}^{T},\ldots ,I_{n}^{T}$} is a dataset containing both normal and abnormal images, where the normal images in $I^{N}$ and $I^{T}$ are independently and identically distributed and the abnormal images in $I^{T}$ are out of distribution. The goal is to train an unsupervised or semi-supervised model to characterize the data in $I^{N}$ and use the model to perform anomaly detection on the test set $I^{T}$, identifying the abnormal images in $I^{T}$. This paper mainly focuses on industrial surface-defect anomalies. Thus, the images used here are mainly images related to the appearance of industrial products. We propose an image anomaly detection model that integrates feature distillation and autoencoder structures, as shown in Figure 1.

The model uses an autoencoder as the main framework, a student network $\Phi_{E}$ obtained from the feature distillation structure of dual-teacher networks as the encoder, and a deconvolution network as the decoder $\Phi_{D}$ to reconstruct the input image. The input image I is fed into both the dual-teacher networks, $\Phi_{T}$, and the student network, $\;\Phi_{E}$, generating a feature-level anomaly map through the discrepancies in features between the student network and the two teacher networks. $\Phi_{E}$ needs to learn the feature representations of two different teacher networks, $\Phi_{T}$, on normal samples. Thus, it needs to acquire more knowledge and find the common information between two feature representations, which helps improve anomaly detection by distillation learning. This paper also proposes an attention mechanism based on extracting the target foreground, generating a mask that can suppress the response to background noise in feature anomaly maps. In addition, an image method for anomaly score calculation based on patch similarity is proposed, which measures the difference between the reconstructed image $I^{\prime }$ constructed by $\Phi_{D}$ and the input image I and generates an image-level anomaly map. Finally, the proposed method combines a feature anomaly map and an image anomaly map to determine whether the input image is abnormal or not.

### 3.1. Dual-Teacher for Knowledge Distillation

STPM is one of the representative methods for anomaly detection based on distillation learning. It adopts the traditional distillation learning structure, using ResNet pre-trained on ImageNet as the teacher network and untrained ResNet as the student network, and lets the student network learn the feature knowledge of the teacher network with normal images. A major problem of STPM lies in the representation of feature differences and anomalies, which may disappear between the student network and the teacher network. This error would result in ineffective differentiation between normal and abnormal images. The dual-teacher network distillation learning structure allows the student network to simultaneously mimic two different teacher networks, thereby “overloading” the student network’s imitation ability and making it more difficult for the student network to reproduce the feature representations of the two teacher networks for the abnormal regions. The student network learns the feature representation of normal images from two teacher networks simultaneously and shares the convolutional features for each layer of the feature maps that need to be distilled. The student network needs to mimic the different convolutional features of the two teacher networks based on the shared feature input. These shared structures make the dual-teacher network more sensitive to the feature representation of anomalies. The student network distills the knowledge from two teacher networks simultaneously, providing multiple perspectives to support it in solving the anomaly detection task. This approach thus helps to improve the accuracy of anomaly detection. Like traditional knowledge distillation methods such as STPM, the proposed dual-teacher network architecture is based on multi-scale feature distillation.

**Figure 2:**
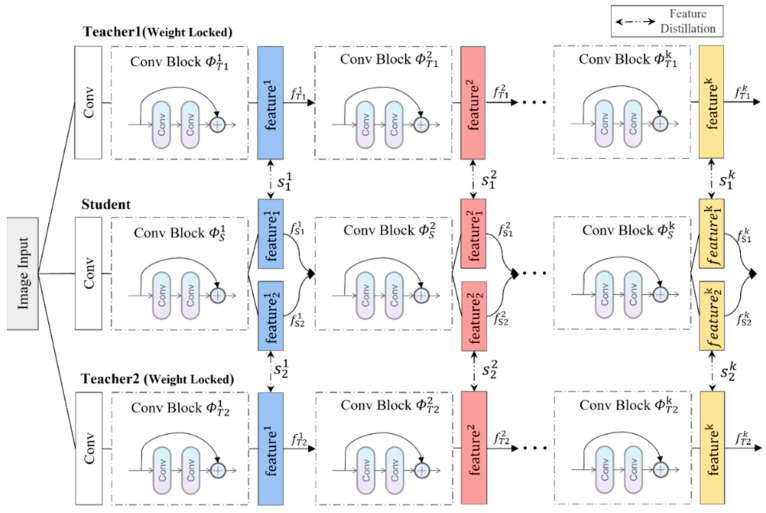
Dual-Teacher network for feature distillation.

The networks can be different types of convolutional neural networks, such as ResNet networks and VGG networks. For convenience in explanation, the following description uses a ResNet network. The dual-teacher network structure is shown in Figure 2. This network structure contains three sub-network branches, two teacher networks and one student network.

In the mathematical description, let I denote the input image, $\Phi_{T1}$ denote the teacher network 1, $\Phi_{T2}$ denote the teacher network 2, and $\Phi_{S}$ denote the student network. Let $\Phi_{T1}^{k}$, $\Phi_{T2}^{k}$, $\Phi_{S}^{k}$ denote the k-th layer module of the teacher network and the student network. Let $f_{T1}^{k}=\Phi_{T1}^{k}\left(f_{T1}^{k-1}\right),\;f_{T1}^{k}=\Phi_{T2}^{k}\left(f_{T2}^{k-1}\right),\;f_{S}^{k}=\Phi_{S}^{k}\left(f_{S}^{k-1}\right)$ express the output of each layer of the three networks, where $f_{T1}^{k},f_{T1}^{k}\in R^{C_{k}\times H_{k}\times W_{k}}$, $f_{S}^{k}\in R^{{2C}_{k}\times H_{k}\times W_{k}}$, 1≤k≤K. K is the number of feature layers that need to be distilled, $C_{k},\;\;H_{k},\;and\;W_{k}$ are the channel number, height and width of the corresponding layer, and $f_{T1}^{0}=f_{T2}^{0}=f_{S}^{0}=I$. The model is described as follows:

1. Construction of teacher networks: Teacher networks with significant structural differences can make it difficult for the student network to learn the shared convolutional layers before the convolutional layer is used for distillation, worsening the model’s convergence. Two networks with the same basic structure (such as two ResNet networks) are used to construct the teacher networks. The networks’ parameters are pre-trained on ImageNet, then used and frozen.
2. Knowledge distillation: In order to obtain high-resolution indications of abnormal positions, cosine similarity or MSE are used as measures of feature vector similarity. The calculation formulas for MSE and cosine similarity are given in Formulas (1) and (2).
  (1) $$\begin{array}{c} {S_{MSE}\left(f_{1},f_{2}\right)}_{\left(h,w\right)}={\left(f_{1}\left(h,w\right)-f_{2}\left(h,w\right)\right)}^{2} \end{array}$$
  (2) $$\begin{array}{c} {S_{COS}\left(f_{1},f_{2}\right)}_{\left(h,w\right)}=1-\frac{{f_{1}\left(h,w\right)}^{T}\cdot f_{2}\left(h,w\right)}{\left\|f_{1}\left(h,w\right)\right\|\cdot \left\|f_{2}\left(h,w\right)\right\|} \end{array}$$
  where $f_{1}$ and $f_{2}$ are two feature maps with the same shape and h and w are the width and height of the feature map, respectively.
3. Multi-scale feature fusion: The distillation information from the two teacher networks and the student network are calculated and fused. The calculation formulas for multi-scale anomaly maps are given in Formulas (3)–(5).
  (3) $$\begin{array}{c} A_{T1}=\sum_{k=1}^{K}{{Upsamle(S}_{MSE/COS}\left(f_{T1}^{k},f_{S1}^{k}\right))}_{\left(H_{1},W_{1}\right)}, \end{array}$$
  $$f_{S1}^{k}={f_{S}^{k}}_{i\;,\;:\;,\;:}\;,0\leq i<\frac{C_{k}}{2}$$
  (4) $$\begin{array}{c} A_{T2}=\sum_{k=1}^{K}{{Upsample(S}_{MSE/COS}\left(f_{T2}^{k},f_{S2}^{k}\right))}_{\left(H_{1},W_{1}\right)}, \end{array}$$
  $$f_{S2}^{k}={f_{S}^{k}}_{i\;,\;:\;,\;:\;},\frac{C_{k}}{2}\leq i<C_{k}$$
  (5) $$\begin{array}{c} A_{DT}=A_{T1}+A_{T2} \end{array}$$
  where ${Upsamle()}_{\left(H_{1},W_{1}\right)}$ is used to upsample the feature map to the shape of the first layer feature map.

After the multi-scale anomaly maps of the two teacher networks are fused, the loss function also adds the maximum value of the fused anomaly map to the distillation model to reduce performance degradation caused by isolated noise. This approach will reduce the occurrence of misjudgments by the model and make it more sensitive to anomalies. The distillation loss of the dual-teacher network is shown in formula (6).

(6) $$\begin{array}{c} L_{AD}=\frac{1}{H_{1}W_{1}}\sum_{h=1}^{H_{1}}\sum_{w=1}^{W_{1}}A_{DT}\left(h,w\right)+\lambda_{M}Max{(A}_{DT}) \end{array}$$

where $\lambda_{M}$ is the weighting factor in $L_{AD}$, used to weight the dual-teacher distillation loss and the maximum suppression loss.

If the structures of the two teacher networks are completely different, the feature information at different scales will have large differences and the student network will have difficulty learning the normal image information. In order to balance the amount of information coming from the two teacher networks, it is recommended that the operator choose two teacher networks that are similar in architecture, such as ResNet18 and ResNet34, VGG16 and VGG19, etc. These teacher networks have similar feature map shapes at the same resolution size. The student network adopts a network structure similar to that of the teacher network. For example, if the teacher networks are ResNet18 and ResNet34, as both teacher networks use BasicBlock as the basic component, then BasicBlock is also used as the basic component when constructing the student network.

### 3.2. ATAA-Abnormal Target Area Attention

One of the problems of anomaly detection methods based on distillation learning is that they cannot distinguish between the object to be detected and the background, and detection performance is easily affected by the background noise. To reduce background noise, a natural idea is create a the network that can generate a mask that can accurately filter out those areas that do not need anomaly detection. This paper proposes integrating a mask-based attention mechanism into the feature difference map that is generated after feature distillation.

The target area usually has richer textural and structural details than the background, so it is hypothesized that the student network has higher learning loss for the target area than for the background. Based on this hypothesis, the ATAA module is proposed.

**Figure 3:**
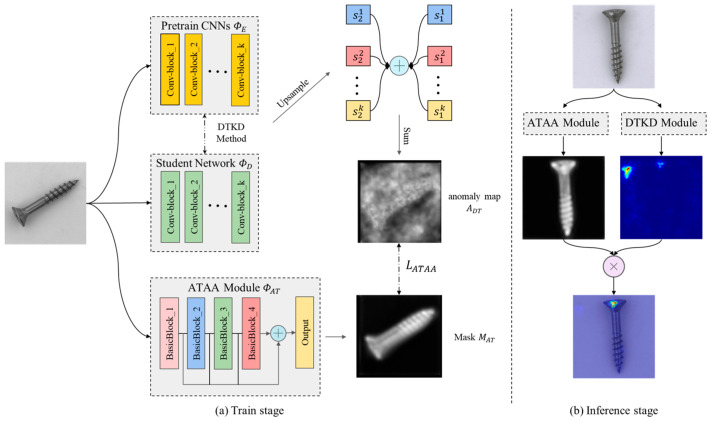
The attention mechanism for highlighting the target objects. The ATAA module learns the regions with high anomaly potential from the distillation loss map during training (**a**) and focuses on product appearance during testing (**b**).

Figure 3 shows in detail how the attention mechanism works in training and inference. Figure 3a shows the process by which the ATAA module learns the areas that are prone to high anomalies in the image during training, where the DTKD method corresponds to the dual-teacher network distillation in Figure 2 and the ATAA module mainly learns from the distillation loss map generated during distillation. Figure 3b shows the process by which the ATAA module handles inference, where the mask generated by ATAA module is multiplied by bit with the anomaly map to filter out the background noise.

The ATAA module takes the image I as input and generates a mask $M_{AT}$ through the convolutional network, and $M_{AT}=\Phi_{AT}\left(I\right)$ is used to represent the attention mask generated by the ATAA module, where $M_{AT}\in R^{C_{1}\times H_{1}\times W_{1}}$. $\Phi_{AT}$ is used to represent the ATAA module. This module is a convolutional network that concatenates four BasicBlocks. After upsampling of the feature maps generated by each BasicBlock to the size of the distillation loss map $A_{DT}$, the feature maps are averaged. The calculation method of $M_{AT}$ is shown in the following formula.

(7) $$\begin{array}{c} M_{AT}=\frac{1}{L}\sum_{l=1}^{L}{Upsample\left(f_{ATAA}^{l}\right)}_{\left(H_{1},W_{1}\right)} \end{array}$$

where $f_{ATAA}^{l}$ represents the feature map generated by the *l*-th BasicBlock in the ATAA module and ${Upsamle()}_{\left(H_{1},W_{1}\right)}$ represents upsampling of the feature map to the shape of $A_{DT}$. The mask $M_{AT}$ will be used as reference information and multiplied by $A_{DT}$ obtained from feature distillation to filter out background noise in $A_{DT}$. The training goal of the ATAA module is to predict the multi-scale distillation loss map generated by each image from the dual teachers during the entire training process. The ATAA module learns the probability distribution of multi-scale distillation loss and abstracts the loss map as a probability distribution, so ATAA’s loss function is the cross-entropy loss between $M_{AT}$ and $A_{DT}$ as shown in the following formula.

(8) $$\begin{array}{c} L_{Pre}=Cross-Entropy\left(sigmoid\left(M_{AT}\right),{A_{DT}}_{no-grad}\right) \end{array}$$

where ${A_{DT}}_{no-grad}$ is the truncating gradient for $A_{DT}$. At the same time, the mask generated by the ATAA module can also guide DTKD to pay more attention to the target area, so an ATAA auxiliary training loss function is added, as shown in following formula:

(9) $$\begin{array}{c} L_{sup}=Mean\left({M_{AT}}_{no-grad}^{norm}*A_{DT}\right)+Max\left((1-{M_{AT}}_{no-grad}^{norm})*A_{DT}\right) \end{array}$$

where ${M_{AT}}_{no-grad}^{norm}$ is the normalizing and truncating gradient for $M_{AT}$.

As ATAA module’s training goal is related to DTKD’s entire training process, the ATAA module’s loss function will have a coupling relationship with the dual-teacher network’s parameters. However, this coupling relationship will greatly increase the dual-teacher network’s training instability, thus causing the entire model’s training to fail. Therefore, in loss functions $L_{Pre}$ and $L_{sup}$, $A_{DT}$ and $M_{AT}$’s gradients are truncated respectively to prevent the coupling relationship from affecting the model’s training. The ATAA module’s complete loss function expression is as follows:

(10) $$\begin{array}{c} L_{ATAA}={\lambda_{A}*L}_{Pre}+\lambda_{s}*L_{sup} \end{array}$$

where $\lambda_{A}$ and $\lambda_{S}$ are the weighting factors in $L_{ATAA}$ and are used to weight the cross-entropy loss and the auxiliary training loss.

The loss function allows the ATAA module to easily and accurately converge on the target area. For those texture class image anomaly detection tasks, although there is no difference between the target object and background, based on the above hypothesis, most structural elements of texture class images are similar on the global image in the ATAA module’s attention mask, which is obtained by calculating texture class images and also tends to be globally distributed.

### 3.3. Anomaly Score Calculation

For the test image $I\in I^{T}$, this paper combines the feature-level anomaly score and the image-level anomaly score to generate the final anomaly score for classification, as follows:

(11) $$\begin{array}{c} S\left(I\right)=S_{DT}\left(I\right)*Patch\text{\_}GMSD\left(I,I_{r}\right) \end{array}$$

where $S_{DT}\left(I\right)$ represents the feature anomaly score produced by the distillation model and Patch_GMSD represents the image anomaly score.

**Figure 4:**
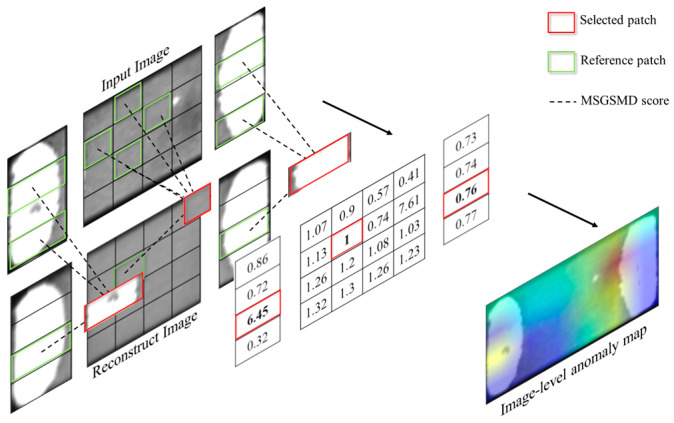
Patch-Based method for anomaly score calculation.

At the image level, to reduce the impact of different-textured regions on the surface on the anomaly score, a patch-based method for anomaly score calculation Patch_GMSD is proposed. Traditional semi-supervised anomaly detection methods based on autoencoders directly calculate the MSE between the reconstructed image and the test image as the anomaly score, but the MSE a relatively poor measure of the similarity between two images because it has weak correlation with human subjective perception. The premise of MSE is that each pixel on the image is independent, but pixels on the image are obviously correlated. In the anomaly score evaluation algorithm, an image quality assessment (IQA) algorithm is obviously more effective than a simple MSE. RIAD improved the GMS algorithm, and the proposed MSGMS algorithm does not consider the abnormal performance of different regions in the image. The abnormal value of the MSGMS map with complex edge information is obviously higher than that of other regions, which can easily cause misjudgment. For this reason, a patch-based method for anomaly score calculation is proposed. The main process of this algorithm is as follows:

1. The image I is divided into *R* regions, and the anomaly score of the image is determined by the maximum anomaly score of the *R* regions. The calculation method is as follows:
  (12) $$\begin{array}{c} Patch\text{\_}GMSD\left(I\right)=max(\lambda_{r}{Patch\text{\_}GMSD}_{r}(I)) \end{array}$$
  where ${Patch\text{\_}GMSD}_{r}$ is the anomaly score of the r-th region and $\lambda_{r}$ is the weight of the r-th region, r ∈ (1, …, R). As different regions have different degrees of reconstruction quality, $\lambda_{r}$ is used to balance the statistical differences between the anomaly scores of different regions. The value of $\lambda_{r}$ is determined by calculating the average Patch_GMSD scores in each region of the training set, such that the average Patch_GMSD scores of the R regions maintain the following relationship to ensure consistency:
  (13) $$\begin{array}{c} avg\left(\lambda_{1}{Patch\text{\_}GMSD}_{1}\left(I\in I^{N}\right)\right)\approx avg\left(\lambda_{2}{Patch\text{\_}GMSD}_{2}\left(I\in I^{N}\right)\right)\approx \cdots \approx avg\left(\lambda_{R}{Patch\text{\_}GMSD}_{R}\left(I\in I^{N}\right)\right) \end{array}$$
2. The r-th image region is divided into $L_{r}^{j}$ patches at each scale j (j ∈ (1, …, J)), and the patch-based anomaly score map is calculated for each scale. The image resolution does not change at different scales, only the method of dividing patches changes. For example, when j = 1, the r-th region is divided into 4 × 4 patches, when j = 2, it is divided into 2 × 2 patches. The anomaly scores of different scales are fused by upsampling, and the maximum value is taken as the anomaly score of the whole region. The calculation of $Patch\text{\_}{GMSD}_{r}$ is as follows:
  (14) $$\begin{array}{c} {Patch\text{\_}GMSD}_{r}={Max}_{t\in P_{r}}\{\sum_{j=1,\ldots J}Upsample(A_{N_{j}\times M_{j}}^{r})\} \end{array}$$
  where $A_{N_{j}\times M_{j}}^{r}$ is the patch-based anomaly score map of the *r*-th region at the *j*-th scale, $N_{j}\times M_{j}$ is the number of patches at that scale, and $N_{j}\times M_{j}=L_{r}^{j}$. Upsample(·) is the upsampling operation, which upsamples $A_{N_{j}\times M_{j}}^{r}$ to the size of $A_{N_{j}\times M_{j}}^{r}$, and $P_{r}\in R^{N_{1}\times M_{1}}$ is the set of patches after upsampling and summation.
3. For the patch at the *j*-th scale, the anomaly score is calculated by calculating the MSGMSD score between the input and reconstructed image patches and the neighboring patches and taking the minimum MSGMSD score as the anomaly score of that patch. As there are more templates for comparison, the misjudgment rate can be reduced. For $A_{N_{j}\times M_{j}}^{r}$, the anomaly score is calculated as follows:
  (15) $$\begin{array}{c} A_{N_{j}\times M_{j}}^{r}\left(h_{n},w_{m}\right)=min\left\{\begin{array}{c} MSGMSD\left(P_{h_{n},w_{m}}^{I},P_{h_{n},w_{m}}^{I^{\prime }}\right),\\ {min}_{h_{a}\in \left\{h_{n}-1,h_{n}+1\right\},w_{a}\in \left\{w_{m}-1,w_{m}+1\right\}}\left\{MSGMSD\left(P_{h_{n},w_{m}}^{I},P_{h_{n},w_{m}}^{I}\right)\right\} \end{array}\right\} \end{array}$$
  where $P_{h_{n},w_{m}}^{I}$ represents the patch at position ($h_{n},w_{m}$) in the input image I, $P_{h_{n},w_{m}}^{I^{\prime }}$ represents the patch at position ($h_{n},w_{m}$) in the reconstructed image $I^{\prime }$, and $h_{n}\in (1,\ldots ,N_{j})$, $w_{m}\in (1,\ldots ,M_{j})$. The MSGMSD score adopts the calculation strategy of the GMSD method, and the calculation formula is as follows:
  (16) $$\begin{array}{c} MSGMSD\left(P,P^{\prime }\right)=\sqrt{\frac{1}{N}\sum_{i=1}^{N}{\left(MSGMS\left(P,P^{\prime }\right)-\frac{1}{N}\sum_{i=1}^{N}MSGMS\left(P,P^{\prime }\right)\right)}^{2}} \end{array}$$
  where P is the input patch and $P^{\prime }$ is the reference patch. *N* represents the number of pixels in the current input patch, and $MSGMSD(P,P^{\prime })$ represents the MSGMS map calculated between the input patch and the reference patch.
4. In the SMDC-DET dataset, SMDC images are predominantly composed of the body and end electrodes. Therefore, taking SMDC images as an example, SMDC images are divided into body regions and two end-electrode regions, with the number of regions set to R = 3. Figure 4 shows the calculation of the anomaly score map of SMDC images at the first scale. When r = 1,3, it represents two end-electrode regions; when r = 2, it represents the body region. As the resolution of SMDC images is 50 × 110, this paper sets only two scales, that is, J = 2. When the number of scales increases, the length of each patch along the *y*-axis will be only 6 pixels and the MSGMSD score cannot be calculated. In the first scale of the end-electrode region, $L_{1}^{1}$ = $L_{3}^{1}\;$ = 4 × 1; in the second scale, $L_{1}^{2}$ = $L_{1}^{3}$ = 2 × 1. In the first scale of the body region, $L_{1}^{2}$ = 4 × 4; in the second scale, $L_{2}^{2}$ = 2 × 2.

The method for calculation of the feature-level anomaly score is as follows: first, the student network is used to generate the anomaly maps $A_{T1}$ and $A_{T2}$ with the two teacher networks respectively, and then the attention mask $M_{AT}$ generated by the ATAA module is used to focus on the abnormal target region. The above calculation process is as follows:

(17) $$\begin{array}{c} S_{DT}\left(I\right)=\max\left({(A}_{T1}*A_{T2})*M_{AT}\right) \end{array}$$

where $M_{AT}$ is the attention mask described in Section 3.2. $A_{T1}$ and $A_{T2}$ are described in Formulas (3) and (4).

### 3.4. The Process of Training

The training process is divided into two stages, the student network training stage and the decoder training stage. After the student network is jointly trained by the dual-teacher network and the ATAA module, it can obtain a compact encoding of the normal image. The loss function of the joint training by the DTKD and the ATAA modules is as follows:

(18) $$\begin{array}{c} L_{DT}=L_{AD}+L_{ATAA} \end{array}$$

where $L_{DT}$ is the loss function of the dual-teacher knowledge distillation and $L_{ATAA}$ is the loss function of the ATAA module.

After the parameters of the student network are fixed, the process enters the decoder training stage. For decoder loss, the model simply uses L1 loss and teacher network content loss as the loss function to guide the optimization of decoder parameters. The structure of the decoder is composed of several deconvolution layers. The decoder loss function is as follows:

(19) $$\begin{array}{c} L_{DEC}={\frac{1}{H\cdot W}\left\|I-I_{r}\right\|}_{L1}+\lambda_{c}\sum_{k=1}^{K}\frac{1}{H_{k}W_{k}}{\left\|f_{T1}^{k}-{\hat{f}}_{T1}^{k}\right\|}_{L2} \end{array}$$

where I is the input image, $I_{r}$ is the reconstructed image, ${\hat{f}}_{T1}^{k}$ is the feature expression of teacher network 1 for the reconstructed image, $f_{T1}^{k}$ is the feature expression of teacher network 1 for the input image, and $\lambda_{c}$ is a weight factor.

## 4. Results

The algorithm test platform for this paper is a server based on the Ubuntu 18.04 system, which uses a 16-core AMD Ryzen 9 5950X processor (Advanced Micro Devices, Inc., Sunnyvale, CA, USA) and a Geforce RTX3090 graphics card with 24 GB of video memory (NVIDIA Corporation, Santa Clara, CA, USA) and 64GB of system memory. The algorithm was subjected to ablation and comparative experiments using the datasets SMDC-DET and MVTec-AD.

### 4.1. Datasets

The SMDC-DET dataset contains surface images of SMD capacitors collected during industrial production, including a rich variety of nine types of defects. The test set contains a total of 462 abnormal images and 1525 normal images, while the training set contains 2456 normal images.

The MVTec-AD dataset is specifically designed for anomaly detection, containing high-resolution images of five textures and ten objects. The test set contains a total of 1258 abnormal images (covering 73 types of defects) and 467 normal images, while the training set contains a total of 3629 training images. In addition, the dataset provides pixel-level labels of abnormal regions for each abnormal image to verify the localization performance of anomaly detection algorithms.

### 4.2. Model Settings

To ensure the computational efficiency of each image, different model settings are adopted for different datasets based on the same algorithmic idea.

For the SMDC-DET dataset, the image input shape (H × W × C) is 128 × 128 × 3, and the teacher network is composed of VGG16 and VGG19, which perform distillation on three feature maps of dimensions 32 × 32 × 128, 16 × 16 × 256, and 8 × 8 × 512. In the SMDC-DET dataset, the image resolution is low, so the VGG series of convolutional neural networks with more parameters were used as the teacher networks. The student network uses a convolutional network similar to the VGG model, which generates three feature maps of dimensions 32 × 32 × 256, 16 × 16 × 512, 8 × 8 × 1024. The decoder reconstructs the image by stacking four deconvolution modules, each consisting of a deconvolution layer, a BN layer and a Relu layer. The feature maps output by each deconvolution module of the decoder are 16 × 16 × 256, 32 × 32 × 128, 64 × 64 × 64, and 128 × 128 × 3 respectively and are followed by a sigmoid layer to normalize the output. An Adam optimizer [37] is used in the training process, with an initial learning rate of 1 × 10^−3^, and batch size set to 32. A total of 400 epochs are trained. To unify the image size, the SMDC images are extracted from the background and uniformly resized to 50 × 110, and black pixels are filled to make the image uniformly 128 × 128 in size. After experimental parameter tuning, the following parameter combination can stabilize the algorithm performance: $\lambda_{M}$ = 2, $\lambda_{s}$ = 2, $\lambda_{A}$ = 0.01, $\lambda_{C}$ = 0.04, and $\lambda_{r}$ = 1 if r = 2 else 0.5, r∈{1, 2, 3}. MSE is used as the measure of feature vector similarity during training.

For the MVTec-AD dataset, the teacher network is composed of Resnet18 and Resnet34, which perform distillation on four feature maps of dimensions 64 × 64 × 64, 32 × 32 × 128, 16 × 16 × 256, and 8 × 8 × 512 generated by Resnet18 and Resnet34′s four residual blocks. In the MVTec-AD dataset, the image resolution is high, so the Resnet series of convolutional neural networks with fewer parameters were used as the teacher networks. The student network uses a ResNet based on BasicBlock residual connections, with four groups of residual blocks, each group consisting of two BasicBlocks in series and generating feature maps of dimensions 64 × 64 × 128, 32 × 32 × 256, 16 × 16 × 512, and 8 × 8 × 1024, respectively. An SGD optimizer is used in the training process, with an initial learning rate of 0.4, momentum set to 0.9, weight decay coefficient of 1 × 10^−4^, and batch size set to 32, and a total of 200 epochs are trained. Due to the numerous types of detection objects in this dataset, there is no targeted region division for each type. Therefore, there is no training decoder and only anomaly scores under feature distillation are used. The algorithm parameters are set as follows: $\lambda_{M}$ = 2, $\lambda_{s}$ = 2, and $\lambda_{A}$ = 0.01, and cosine similarity is used as the measure of feature vector similarity during training.

### 4.3. Evaluation Metrics

The AUROC score is used as the evaluation metric for anomaly detection performance. AUROC draws an ROC curve by selecting different thresholds and calculates the area under the curve as the AUROC score. The closer the AUROC score is to 1, the better the model can distinguish between normal and abnormal images. The ROC curve is a curve drawn with the false positive rate (FPR) as the horizontal axis and the true positive rate (TPR) as the vertical axis for different thresholds. FPR and TPR are defined as follows:

(20) $$\begin{array}{c} FPR=\frac{TP}{TP+TN} \end{array}$$

(21) $$\begin{array}{c} TPR=\frac{FP}{TN+FP} \end{array}$$

where TP is the number of samples that are predicted to be positive and actually are positive, TN is the number of samples that are predicted to be negative and actually are negative, and FP is the number of samples that are predicted to be positive but actually are negative.

### 4.4. Comparative Experiment

For the SMDC-DET dataset, the task objective is anomaly detection at the image level. The proposed algorithm is compared with previous methods, such as STPM [28], f-anoGAN [38], cutpaste [39], MKD [9], RIAD [23], and SPADE [40]. The experimental results are shown in Table 1. From Table 1, the proposed method achieves an AUC score of 98.85%, an FPR of 5.17% when TPR is fixed at 95%, and a TPR of 96.88% when FPR is fixed at 10%. Compared with other distillation or reconstruction methods, it shows significant improvement, which verifies the effectiveness of the method that combines a distillation model and a generative model.

For the MVTec-AD dataset, both anomaly detection and localization tasks need to be completed. The AUROC score is used as the metric for both anomaly detection and anomaly localization. The comparison methods are STPM [28], US [27], MKD [9], cutpaste [39], RIAD [23], SPADE [40], and Patch-SVDD [41]. The experimental results are shown in Table 2 and Table 3.

Table 2 shows the anomaly detection results. From Table 2, the proposed DTKD method achieves the best results on most classes at the image level on the MVTec-AD dataset. For texture and object anomaly detection, the average performance of DTKD reaches 99.6% and 98.6%, respectively, and the overall average on the MVTec-AD dataset reaches 98.9%, which is very advanced performance.

Table 3 shows the pixel-level results. From Table 3, it can be seen that this method performs best among the multiple-comparison methods tested. The average AUC score on texture classes is 97.4%, and the average AUC score on object classes is 97.9%. The overall average AUC score is 97.7%.

### 4.5. Ablation Experiments and Analyis

Detailed ablation experiments were conducted to study the proposed modules and methods. For different datasets, different baselines were chosen to verify the effectiveness of the modules and model structures due to the differences in the model backbone and anomaly score settings.

For the SMDC dataset, AE-GMSD was chosen as the baseline for ablation experiments. The AE structure used the student network and decoder directly, used MSGMS as the anomaly score, and conducted experiments on the following modules and methods: (1) DTKD, (2) ATAA, and (3) $Patch\text{\_}{GMSD}_{SMD}$. For the MVTec-AD dataset, STPM was chosen as the baseline. Experiments were conducted on the following modules and methods: (1) DTKD and (2) ATAA. The experimental results are shown in Table 4.

From Table 4, it can be seen that the proposed modules and structures improved the AUC score performance on the SMDC dataset. For AE, due to hypothetical flaws in this structure, the AUC score was only 87.25%, while according to the anomaly score, $Patch\text{\_}{GMSD}_{SMD}$ measurement improved the performance of AE model by 8.88%, to 96.13%. With embedded MKD structure, using $Patch\text{\_}{GMSD}_{SMD}$ measurement alone improved the model performance to 97.24%. In the ablation experiment on the distillation network, under the dual-teacher network structure, DTKD’s distillation model performance improved to 95.17% compared to MKD, while the ATAA module did not show significant improvement when used on the SMDC dataset. The specific causes of this difference will be elaborated in detail later. Finally, with the comprehensive combination of multiple modules and structural methods, the overall performance of the model reached 98.85%, according to the AUC score.

For the MVTec-AD dataset, the ablation experiments of the modules and structures are shown in Table 5. Compared with the baseline algorithm STPM, DTKD improved the average AUC score by 2.3, reaching an average AUC score of 97.8%. This result shows the effectiveness of the dual-teacher network structure. Based on ATAA’s performance, it can be improved by 1%, allowing the model to reach 98.9% when it is used on the MVTec-AD dataset.

**Figure 5:**
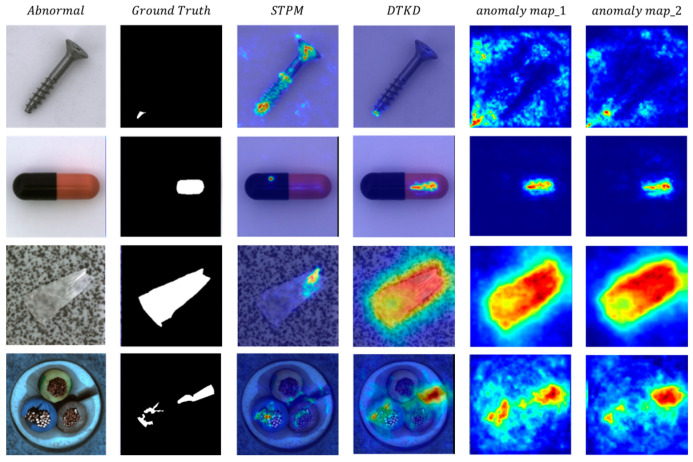
Comparison of single-teacher and dual-teacher networks used on the MVTec-AD dataset. The dual-teacher network can produce more accurate anomaly maps than the single-teacher network. The dual-teacher network also generates two anomaly maps that are multiplied to obtain a fused anomaly map with error-correction ability.

1. Analysis of the effectiveness of DTKD:

Table 4 and Table 5 show the detailed results of ablation experiments conducted with DTKD, which improved the AUC score from 94.33 to 95.17 when used on the SMDC dataset compared to the traditional method, MKD, and improved the Image-Level AUC score by 2.3 for the MVTec-AD dataset compared to the baseline method, STPM. This result demonstrates that the DTKD strategy can improve anomaly detection over traditional distillation methods. It is more difficult for the student network to reproduce the knowledge representation of both teacher networks for abnormal regions, resulting in a more sensitive response to abnormal regions. To explore the advantages of the dual-teacher network over a single-teacher network, we visualized the difference between a single-teacher network and a dual-teacher network in their response to abnormal images. Figure 5 shows the anomaly maps of a single-teacher network and a dual-teacher network for the same sample. It can be seen that the two anomaly maps obtained by the dual-teacher network have some error correction ability because the maps can be multiplied them together to capture possible abnormal regions from multiple perspectives. In the tile, capsule, and cable categories in Figure 5, the student network and the teacher network of STPM produced similar feature representations for some abnormal regions, which weakened the model’s ability to judge anomalies. However, for the same regions, DTKD showed more sensitive responses.

2. Analysis of the effectiveness of ATAA

The design purpose of the ATAA module is to help solve the problem that anomaly detection methods based on knowledge distillation, such as STPM and MKD, are easily affected by background noise, which affects the results. The ablation experiment results in Table 4 show that the effect of using the ATAA module on the SMDC-DET dataset is not obvious because background replacement was performed on the SMDC-DET images during data preprocessing, so background noise was eliminated. Table 5 shows that the ATAA module can increase the average AUC score of the DTKD algorithm on the MVTec-AD dataset to 98.9%. The improvement is mainly concentrated on object-level anomaly detection for objects such as a screw, toothbrush, capsule, and pill, which are categories for which knowledge distillation algorithms need to be improved. Table 6 shows the performance improvement brought by the ATAA module at the image level for these categories.

**Figure 6:**
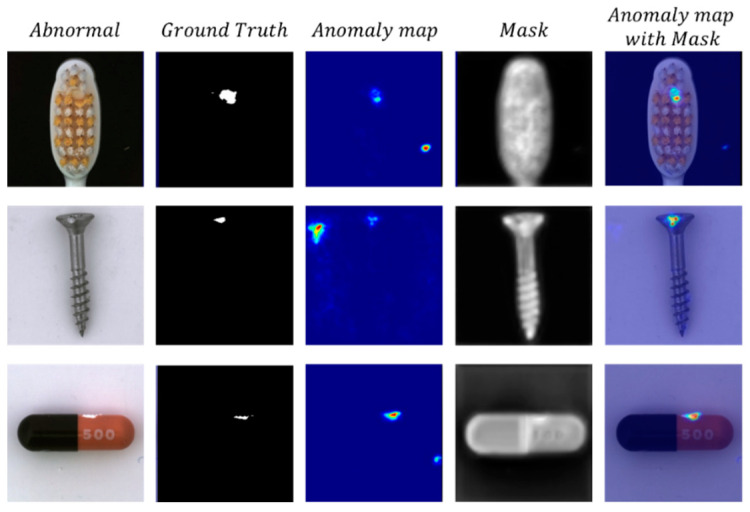
The effect of the ATAA module on the anomaly maps. The ATAA module generates a mask to reduce background noise.

Figure 6 shows the auxiliary role of the ATAA module in filtering out noise for DTKD. Without the ATAA module, DTKD may respond to abnormal areas outside the target area, but the ATAA module can help DTKD focus on areas where abnormalities may occur.

3. Analysis of the effectiveness of the distillation-decoding structure

**Figure 7:**
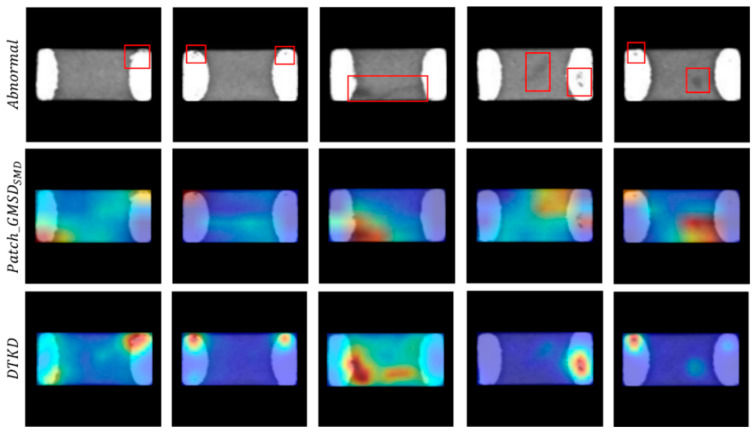
The difference between feature-level and image-level anomaly responses. The red square in the figure represents the defect location in the SMDC image. The combination of the two strategies can help to improve the model’s sensitivity to anomalies and provide multiple perspectives for anomaly detection.

In the method proposed for the SMDC dataset, in addition to anomaly detection by knowledge distillation, the last 8 × 8 feature map of the student network is used as input to the decoder, providing another dimension of analysis for the feature information of the student network. The ablation experiment represented in Table 4 shows that after the DTKD and decoder reconstruction of image-level information are combined for comparison, the AUC of the proposed model improved to 98.85% on the SMDC-DET dataset. Figure 7 shows the difference between feature-level and image-level responses for a single sample. It can be seen that for some abnormal areas, combining two strategies can help improve the model’s sensitivity to abnormalities.

4. Analysis of the $Patch\text{\_}{GMSD}_{SMD}$ method

**Figure 8:**
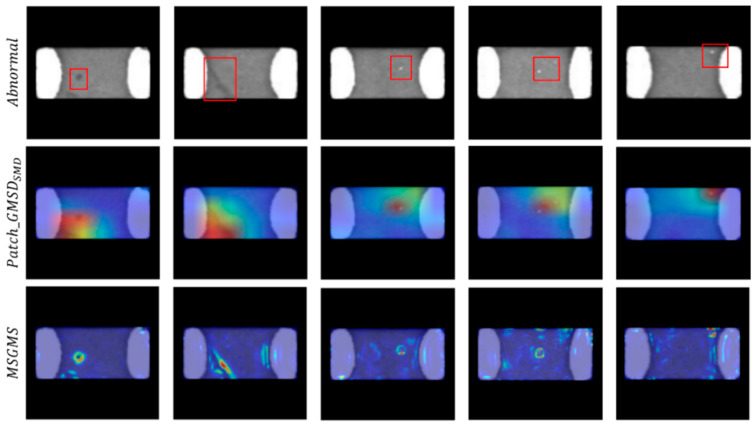
The effect of the patch-based method for anomaly score calculation on the anomaly maps. The red square in the figure represents the defect location in the SMDC image. The proposed method can better reflect the structural and texture differences of the images and improve the sensitivity and accuracy of the anomaly score.

$Patch\text{\_}{GMSD}_{SMD}$ is the Patch_GMSD method for SMDC-like structures. In the experiment shown in Table 4, when the anomaly score is added to the $Patch\text{\_}{GMSD}_{SMD}$ score, the model achieves 98.85 AUC on the SMDC dataset. It is worth noting that when the model degenerates into a single autoencoder, the autoencoder model using the MSGMS method achieves only 87.25% AUC on the SMDC dataset due to the irregularity of electrode edges and the incompleteness of autoencoder reconstruction. The $Patch\text{\_}{GMSD}_{SMD}$ method can improve the AUC score to 96.13%. The responses of the two methods to abnormal samples are shown in Figure 8. Although the patching step of $Patch\text{\_}{GMSD}_{SMD}$ leads to a low-resolution result, this strategy is very helpful for filtering out noise, and each patch calculates the MSGMSD score with its surrounding patches, an approach that can avoid the disadvantage of AE reconstruction, which is that the reconstructed image may not be similar to the original. The purpose of calculating the MSGMSD score separately for the electrode area and the body area is to avoid the uncertainty caused by the irregularity of the electrode edge. Figure 8 shows that these steps can help $Patch\text{\_}{GMSD}_{SMD}$ respond to most of the anomalies while effectively suppressing the noise caused by high-frequency details such as edges.

Table 7 shows the impact of using different strategies in $Patch\text{\_}{GMSD}_{SMD}$ on the SMDC-DET dataset. In this experiment, the baseline strategy is to directly calculate the MSGMSD score between the input image and the reconstructed image at the image level. From Table 7, it can be seen that the region-division strategy results in the most significant improvement in performance; the neighboring-patches comparison strategy provides more reference information for the local comparison with the input image; and the multi-scale strategy results in better detection of defects at different scales.

## 5. Conclusions

This paper proposes an image anomaly detection method based on feature distillation and autoencoder structure that can effectively use pre-trained convolutional neural networks to extract normal image features and enhance the student network’s sensitivity to anomalies through dual-teacher network and ATAA module. The method also introduces a patch-based method for anomaly score calculation, which can overcome the limitations of MSE and other traditional indicators and better reflect the structural and texture differences in images. The authors of this paper conducted experiments on two datasets, verifying the effectiveness and superiority of the proposed method. The experimental results are as follows: on the MVTec-AD dataset, the proposed method achieves an average AUC score of 98.9%; on the SMDC-DET dataset, the proposed method achieves an average AUC score of 98.8%. In summary, the proposed method shows excellent performance on various types of image anomaly detection tasks, proving its good generalizability and robustness. This paper provides a novel and effective method for image anomaly detection.

## Figures and Tables

**Table 1:** Image-level AUC scores of different methods used on the SMDC-DET dataset.

Method	Type	FPR (TPR = 95%)	TPR (FPR = 10%)	AUC (%)
RIAD	Reconstruction	71.63	41.56	76.83
SPADE	Embedding similarity	57.58	60.98	84.72
f-anoGAN	Reconstruction	54.82	64.51	87.28
Cutpaste	Data augmentation	46.45	73.37	90.42
STPM	Distillation	48.57	76.88	91.17
MKD	Distillation	32.13	82.68	94.33
Ours	Reconstruction + Distillation	5.17	96.88	98.85

**Table 2:** Image-level AUC scores of different methods used on the MVTec-AD dataset.

Category		Patch-SVDD	US	STPM	MKD	Cutpaste	RIAD	SPADE	Ours
Textures	Carpet	92.9	91.6	-	79.3	93.9	84.2	-	99.3
	Grid	94.6	81.0	-	78.0	100.0	83.3	-	99.9
	Leather	90.9	88.2	-	95.1	100.0	88.5	-	99.9
	Tile	97.8	99.1	-	91.6	94.6	84.5	-	99.2
	Wood	96.5	97.7	-	94.3	99.1	100	-	99.6
	Average	94.5	91.5	-	87.6	97.5	93.0	-	99.6
Objects	Bottle	98.6	99.0	-	99.4	98.2	99.9	-	100.0
	Cable	90.3	86.2	-	89.2	81.2	88.4	-	98.6
	Capsule	76.7	86.1	-	80.5	98.2	99.6	-	98.1
	Hazelnut	92.0	93.1	-	98.4	98.3	100	-	100.0
	Metal Nut	94.0	82.0	-	73.6	99.9	83.8	-	99.6
	Pill	86.1	87.9	-	82.7	94.9	98.7	-	96.8
	Screw	81.3	54.9	-	83.3	88.7	90.9	-	97.6
	Toothbrush	100	95.3	-	92.2	99.4	98.1	-	97.2
	Transistor	91.5	81.8	-	85.6	96.1	81.9	-	98.9
	Zipper	97.9	91.9	-	93.2	99.9	84.2	-	98.8
	Average	90.8	85.8	-	87.8	95.5	83.3	-	98.6
Total Average		92.1	87.7	95.5	87.7	96.1	91.7	85.5	98.9

**Table 3:** Pixel-level AUC scores of different methods used on the MVTec-AD dataset.

Category		Patch-SVDD	US	STPM	MKD	Cutpaste	RIAD	SPADE	Ours
Textures	Carpet	92.6	93.5	-	95.64	98.3	96.3	97.5	98.7
	Grid	96.2	89.9	-	91.78	97.5	98.8	93.7	98.9
	Leather	97.4	97.8	-	98.05	99.5	99.4	97.6	99.1
	Tile	91.4	92.5	-	82.77	90.5	89.1	87.4	95.2
	Wood	90.8	92.1	-	84.8	95.5	85.8	88.5	94.9
	Average	93.7	93.2	-	90.61	96.3	93.9	92.9	97.4
Objects	Bottle	98.1	97.8	-	96.32	97.6	98.4	98.4	98.6
	Cable	96.8	91.9	-	82.4	90.0	84.2	97.2	96.9
	Capsule	95.8	96.8	-	95.86	97.4	92.8	99.0	98.9
	Hazelnut	97.5	98.2	-	94.62	97.3	96.1	99.1	98.9
	Metal Nut	98.0	97.2	-	86.38	93.1	92.5	98.1	98.5
	Pill	95.1	96.5	-	89.63	95.7	95.7	96.5	99.1
	Screw	95.7	97.4	-	95.96	96.7	98.8	98.9	99.2
	Toothbrush	98.1	97.9	-	96.12	98.1	98.9	97.9	98.8
	Transistor	97.0	93.0	-	76.45	93.0	87.7	94.1	91.4
	Zipper	95.1	95.6	-	93.9	99.3	97.8	96.5	98.5
	Average	96.7	94.3	-	90.76	95.8	94.3	97.6	97.9
Total Average		95.7	93.9	97.0	90.71	96.0	94.2	96.5	97.7

**Table 4:** Results of ablation experiments for different modules tested on the SMDC-DET dataset.

MKD	DTKD	ATAA	$Patch\text{\_}{GMSD}_{SMD}$	AUC (%)
×	×	×	×	87.25
√	×	×	×	94.33
×	√	×	×	95.17
×	√	√	×	95.21
×	×	×	√	96.13
√	×	×	√	97.24
×	√	√	×	96.12
×	√	√	√	98.85

**Table 5:** Results of ablation experiments on different modules used on the MVTec-AD dataset.

STPM	DTKD	ATAA	AUC (%)
√	×	×	95.5
×	√	×	97.8
×	√	√	98.9

**Table 6:** The effect of using the ATAA module on some object classes in the MVTec-AD dataset.

Object	STPM (without ATAA)	STPM (with ATAA)	DTKD (without ATAA)	DTKD (with ATAA)
Screw	88.2	92.2	94.1	97.6
brushtooth	87.8	97.0	88.3	97.2
capsule	88.0	84.0	95.6	98.1
Pill	93.8	95.0	95.8	96.8

**Table 7:** The effect of using various strategies in $Patch\text{\_}{GMSD}_{SMD}$ on the SMDC-DET dataset.

Region Division	Neighboring Patches Comparison	Multi-Scale	AUC(%)
×	×	×	96.12
√	×	×	97.52
√	√	×	98.33
√	√	√	98.85

## Data Availability

Data are contained within the article.
